# Visualizing temporal dynamics and research trends of macrophage-related diabetes studies between 2000 and 2022: a bibliometric analysis

**DOI:** 10.3389/fimmu.2023.1194738

**Published:** 2023-07-26

**Authors:** Sicheng Wang, Lili Zhang, Zishan Jin, Yayun Wang, Boxun Zhang, Linhua Zhao

**Affiliations:** ^1^ Institute of Metabolic Diseases, Guang’ Anmen Hospital, China Academy of Chinese Medical Sciences, Beijing, China; ^2^ Graduate School, Beijing University of Chinese Medicine, Beijing, China; ^3^ Graduate School, Changchun University of Chinese Medicine, Jilin, China

**Keywords:** macrophage, diabetes mellitus, macrophage polarization, bibliometric analysis, visualization

## Abstract

**Background:**

Macrophages are considered an essential source of inflammatory cytokines, which play a pivotal role in the development of diabetes and its sequent complications. Therefore, a better understanding of the intersection between the development of diabetes and macrophage is of massive importance.

**Objectives:**

In this study, we performed an informative bibliometric analysis to enlighten relevant research directions, provide valuable metrics for financing decisions, and help academics to gain a quick understanding of the current macrophage-related diabetes studies knowledge domain.

**Methods:**

The Web of Science Core Collection database was used for literature retrieval and dataset export. Bibliometrix R-package was performed to conduct raw data screening, calculating, and visualizing.

**Results:**

Between 2000 and 2022, the annual publication and citation trends steadily increased. Wu Yonggui was the scholar with the most published papers in this field. The institute with the highest number of published papers was the University of Michigan. The most robust academic collaboration was observed between China and the United States of America. *Diabetologia* was the journal that published the most relevant publications. The author’s keywords with the highest occurrences were “inflammation”, “diabetic nephropathy”, and “obesity”. In addition, “Macrophage polarization” was the current motor topic with potential research prospects.

**Conclusions:**

These comprehensive and visualized bibliometric results summarized the significant findings in macrophage-related diabetes studies over the past 20 years. It would enlighten subsequent studies from a macro viewpoint and is also expected to strengthen investment policies in future macrophage-related diabetes studies.

## 1 Introduction

The prevalence of diabetes is skyrocketing across the globe, which has become a severe public health issue. According to current estimates from International Diabetes Federation, diabetes is a metabolic disorder affecting approximately 537 million adults in 2021, an astonishing increase of 74 million (16%) compared with 2019 (1). Type 2 diabetes(T2D) is the most common type, characterized by insulin resistance and progressive β-cell dysfunction, which gradually deteriorates glucose homeostasis from prediabetes to diabetes. It can be attributed to oxidative stress, ectopic lipid deposition, lipotoxicity, and glucotoxicity leading to long-term micro and macrovascular complications (2–4). Worth noting that each of these potential mechanisms is linked to inflammation (5). A meta-analysis based on prospective studies has revealed that elevated levels of C-reactive protein (relative risk: 1.26 [95% CI, 1.16 to 1.37]) and interleukin-6 (IL-6) (relative risk: 1.31 [95% CI, 1.17 to 1.46]) are associated with an increased risk of T2D (6). Autoimmunity is the hallmark of type 1 diabetes—this multifactorial, organ-specific autoimmune disease results from insulitis by infiltrating immune cells. During insulitis, effector T cells secrete high levels of proinflammatory cytokines like tumor necrosis factor-alpha (TNF-α), IL-1*β*, and IL-12, which trigger the process of *β* cell destruction (7, 8).

It is widely accepted that multiple immune cell types participate in initiating and maintaining metabolic disorders, including diabetes. Macrophages are considered an essential source of inflammatory cytokines, which play a pivotal role in the development of diabetes and its sequent complications (9, 10). Furthermore, macrophage activation has been commonly used to describe macrophage activity in response to diverse stimuli that include microbial products, damaged cells, and particularly inflammatory cells (11). If the inflammatory conditions are not properly dealt with, it may result in the persistent activation of the immune system, which can contribute to the development of diabetes. However, macrophages are highly plastic, indicating that distinct functional subsets undergoing different phenotypic differentiation can exert their unique biological effects, including anti-inflammatory actions. Therefore, to discover and promote new molecular targets and therapies to improve life expectancy, a better understanding of the intersection between the development of diabetes and macrophage is of massive importance. Notwithstanding, there is no comprehensive elucidation of the current state and research hotspots of macrophage-related diabetes studies (MRDS).

In 1969, Pritchard defined bibliometric analysis as “the application of mathematical and statistical methods to books and other media of communication”. As a quantitative science approach that monitors and evaluates research characteristics and trends based on published data within a specific timeframe, it has gained popularity in medical research (12, 13). Based on powerful algorithms, Bibliometrix R-package is a widely used open-source science mapping application with solid visualization and data analysis capabilities (14). In this study, we performed a bibliometric analysis to inform relevant research directions, provide valuable metrics for financing decisions, and gain a quick understanding of the MRDS knowledge domain for academics through the following steps with visualization:(1) Calculate growth trends of publications and citations; (2) Discover collaboration between core countries, authors, and institutions; (3) Investigate influential journals and top-cited publications; (4) Summarize dynamic changes of research hotspots and detect forefront topics.

## 2 Materials and methods

### 2.1 Retrieval strategy and dataset establishment

As one of the primary sources for scientometric analysis, the Web of Science (WoS) has a well-established citation network in different research fields (15). Dr. Ho Yuh-Shan proposed that the WoS topic search (TS) result in the intersectional knowledge domain may not be consistent with the article’s theme (16). Therefore, to get our bibliometric data more precisely, a systematic search strategy was developed using the following search terms based on title (TI) and author’s keyword (AK) search in the Science Citation Index Expanded (SCIE) of the WoS core collection database, regardless of language or document type: (TI=(diabet*) OR AK=(diabet*)) AND (TI=(macrophag*) OR AK=(macrophag*)). Notably, the raw data of publication records were extracted on a single day (December 4th, 2022) to avoid deviation errors from the WoSCC and saved as a TXT file.

**Figure 1:**
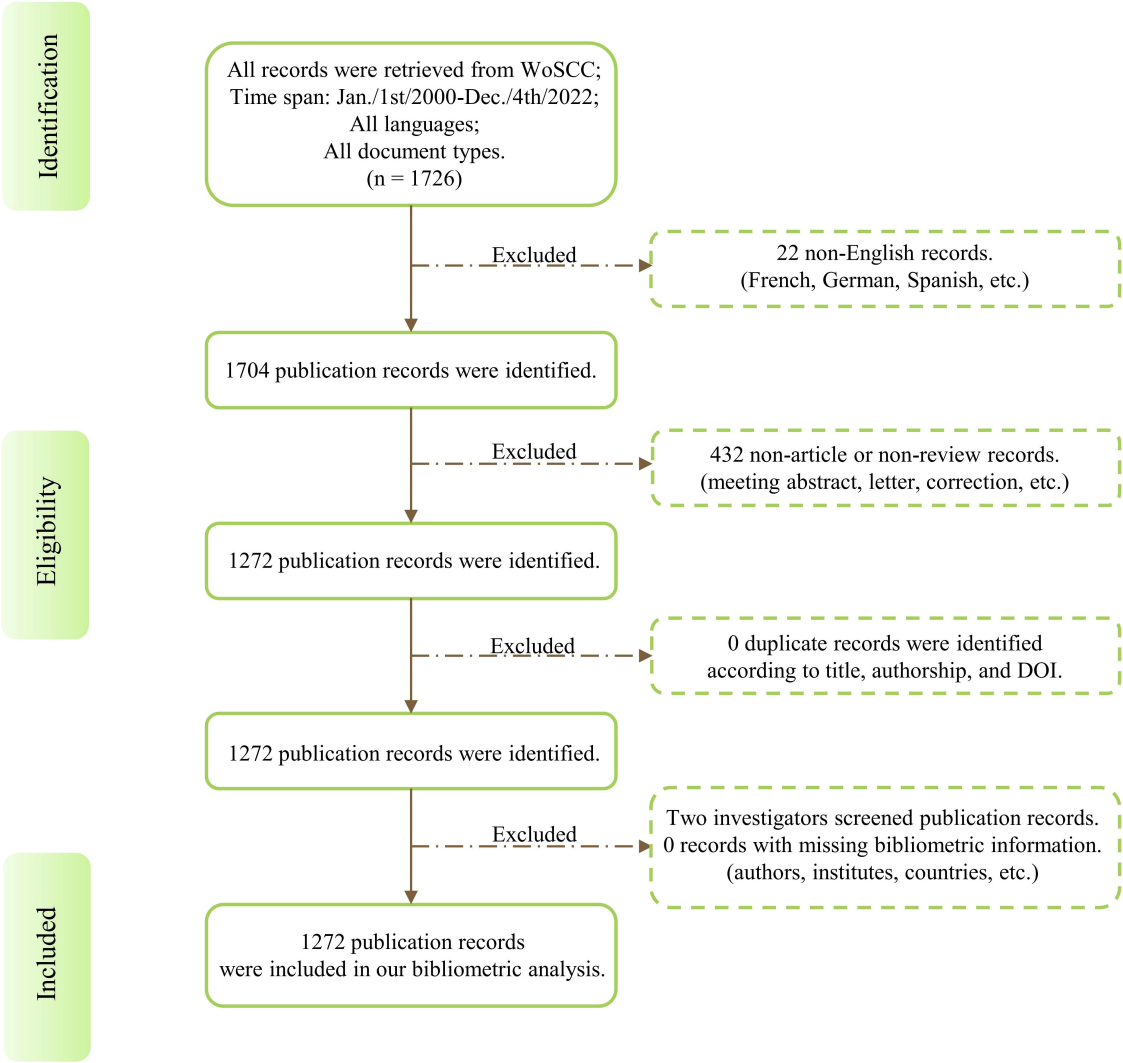
Flow chart of bibliometric analysis dataset establishment. DOI: Digital Object Unique Identifier.

Bibliometrix (version 3.2.1, R version 4.2.0) was performed to conduct raw data screening by two independent investigators. Publications fulfilling the following criteria would be included in our bibliometric analysis: (1) Papers that were published in English; (2) Document type should be review or article; (3) Papers that had no duplicates according to the authorship, publication title, and DOI number. Otherwise, only one of all duplicates would be retained;(4) Publication records must have complete information on authors, institutes, countries, journals, keywords, and citations. Two investigators (S.W. and Z. J.) independently screened each publication record for eligibility, and any discrepancies were settled through consultation with other authors to obtain a more precise data selection. The detailed filtering process is shown in **Figure 1**.

### 2.2 Data analysis and visualization

Bibliometrix R-package was applied to perform statistical and visualizing procedures. In our study, we analyzed and summarized annual publications and citations, the contribution of authors, institutes, countries, journals, and keywords to explore research trends, collaborations, reliable sources, internal structures of research hotspots, and temporal distribution of frontier topics.

Specifically, the Hirsch index (H-index), an acknowledged quantitative metric defined as the maximum value of H such that an author has H publications that have each been cited at least H times, was performed to measure the academic influence of authors (17). The strength of journal influence was assessed by Impact Factor, which was obtained from the 2021 Journal Citation Report (JCR) (http://mjl.clarivate.com/). PageRank score, proposed by Larry Page, the Google company sponsor, is an alternative and newly emerged measurement of academic impact calculated by computer-based algorithms (18, 19). It was designed to embody the relevance and importance of different web pages. In our network analysis, those with a high PageRank score implied more intensive academic connections. Our previous work has proven this quantitative metric more intuitive and efficient (20).

## 3 Results

### 3.1 Temporal distribution of publications and citations

**Figure 2:**
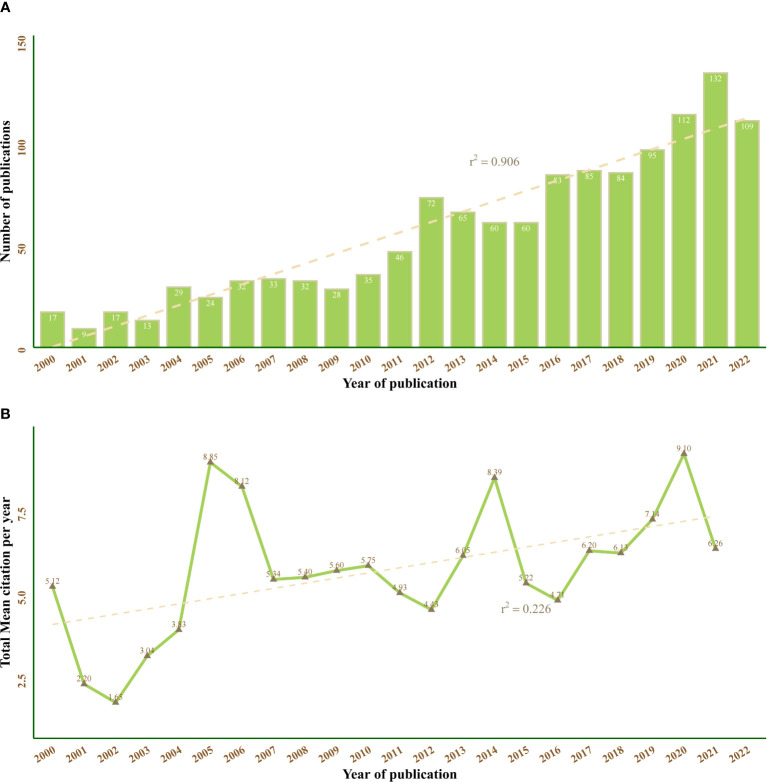
Annual trend chart of publications and citations between 2000 and 2022. The dotted line represents linear growth with r^2 = ^0.906 **(A)** and r^2 = ^0.226 **(B)**.

A total of 1272 publications were selected in our bibliometric study, including 1099 original research full-length articles and 173 reviews. An apparent trend in annual publication numbers was observed in the past two decades, indicating a skyrocketed interest of endocrinologists in macrophage research (**Figure 2A**). Specifically, this growth could be divided into three phases: from 2000 to 2011 was the beginning study phase with less than 50 publications annually. Then, a stable growth period was found between 2012 to 2015. The number of publications has entered a tremendous development phase from 2016 onwards and peaked at 132 in 2021. The total mean citation per year represents the yearly average number of times each publication of MRDS has been cited. This metric can reveal the overall picture of the academic impact of papers published in a certain year. As shown in **Figure 2B**, literature published in 2005, 2006, 2014, and 2020 with higher citation times.

### 3.2 Visual analysis of active authors

**Figure 3:**
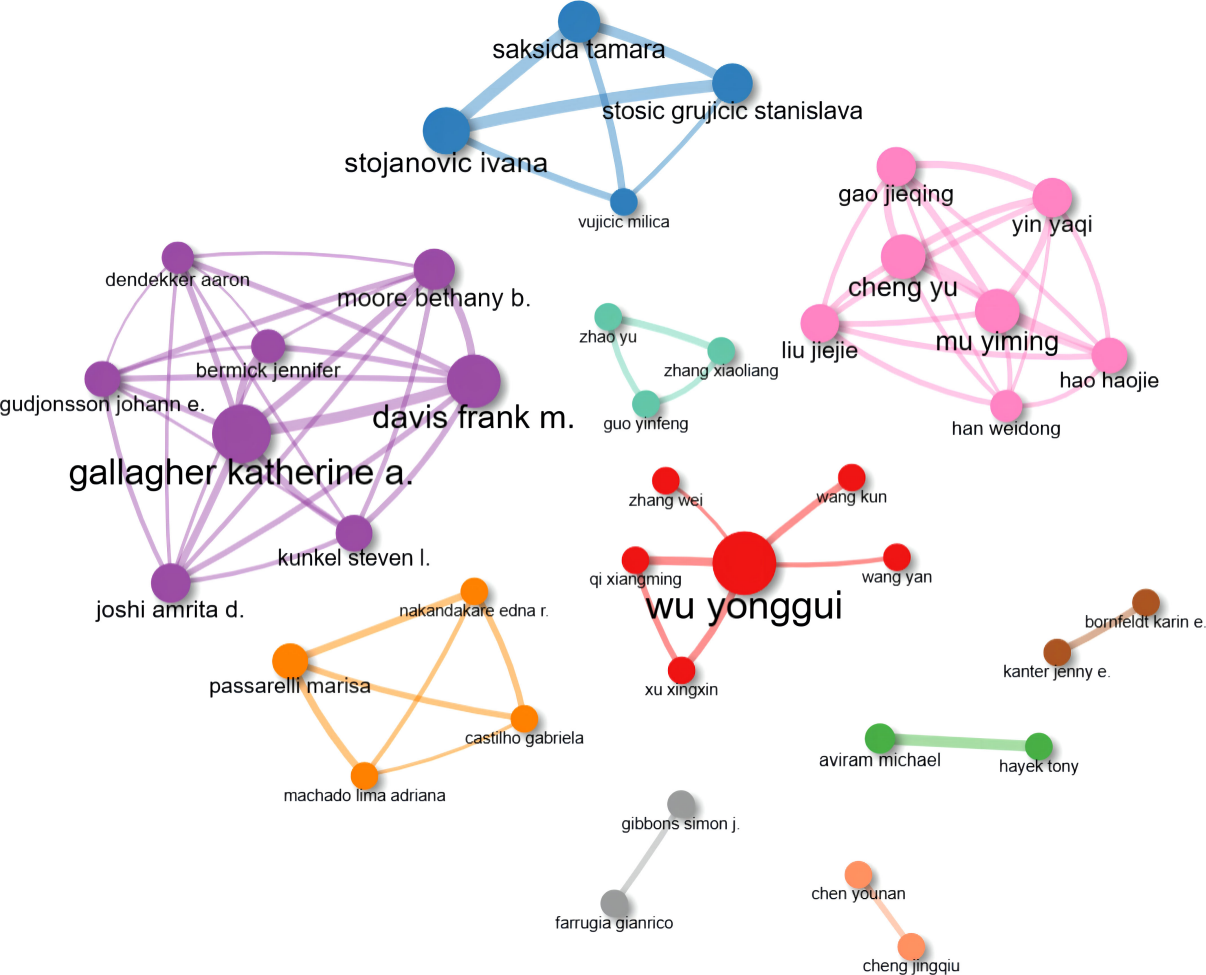
Collaboration network map of the top 50 most prolific authors. Nodes represent different authors, and lines represent connections between them. Ten isolated nodes were removed.

In the past two decades, a total of 7520 authors have made their contributions to MRDS. **Table 1** listed the top ten most prolific authors: Wu Yonggui was the scholar with the most published papers in this field (23 publications), followed by Stojanovic Ivana (12 publications), Aviram Michael (11 publications), and Gallagher Katherine A. (11 publications). In terms of H-index, Wu Yonggui (H-index:12), Aviram Michael (H-index:10), and Gallagher Katherine A. (H-index:9) and Stojanovic Ivana (H-index:9) ranked in the top three. The collaborations among the top 50 most prolific authors are shown in **Figure 3**. A total of eight academics, centered on Gallagher Katherine A. (PageRank score: 0.034), constituted the largest collaborative cluster of authors (purple group).

**Table 1:** Top ten most prolific authors between 2000 and 2022.

Ranking	Author	H- Index	TC	Count (% of 1272)	PageRank score
1	Wu Yonggui	12	393	23 (1.81%)	0.060
2	Stojanovic Ivana	9	198	12 (0.94%)	0.029
3	Aviram Michael	10	663	11 (0.86%)	0.025
4	Gallagher Katherine A.	9	460	11 (0.86%)	0.034
5	Passarelli Marisa	8	198	10 (0.79%)	0.029
6	Saksida Tamara	8	120	10 (0.79%)	0.027
7	Stosic Grujicic Stanislava	8	187	10 (0.79%)	0.025
8	Davis Frank M.	7	228	9 (0.71%)	0.032
9	Zhang Wei	6	170	9 (0.71%)	0.010
10	Hayek Tony	8	495	9 (0.71%)	0.025

TC represents total citations.

**Figure 4:**
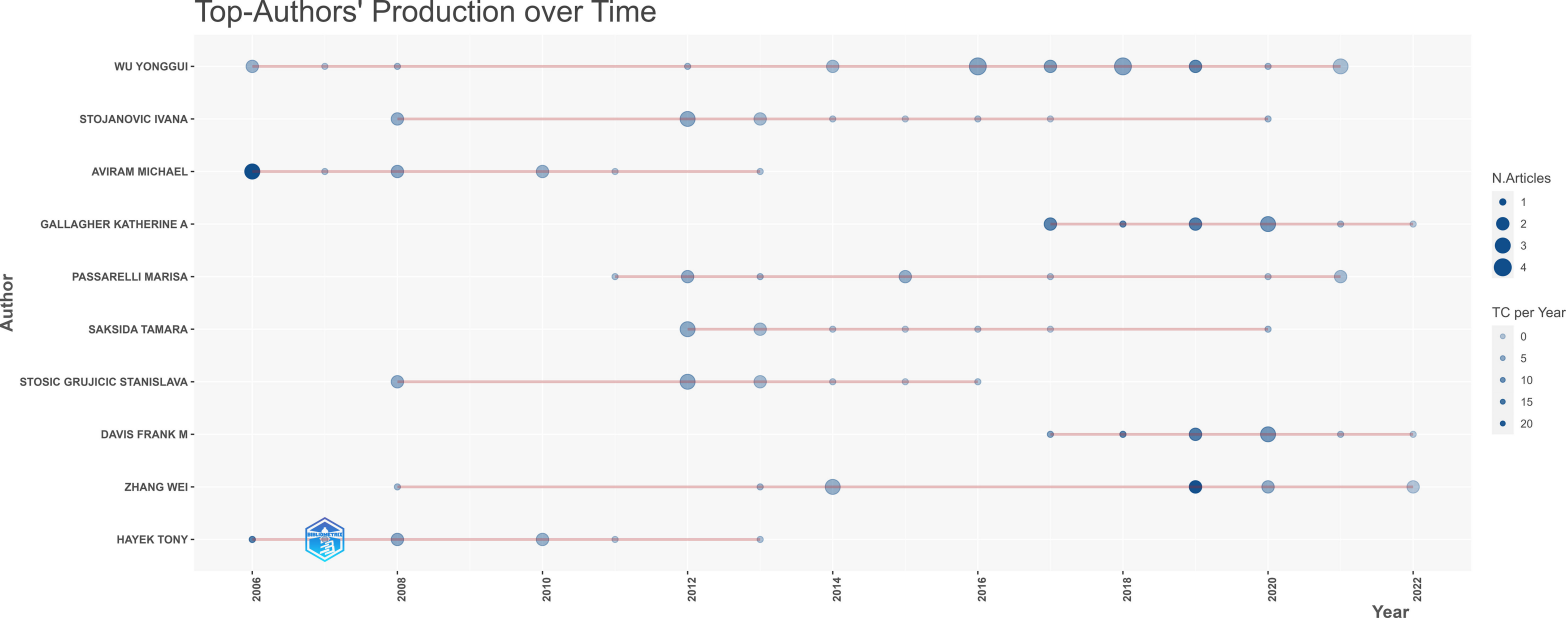
Publications and total citations per year of the top ten prolific authors between 2000 and 2022. the size of the circles represents the number of publications, and the color of the circles represents the number of citations per year.

Additionally, we summarized the annual publication numbers and citation times of the top ten high-yielding authors, and Gallagher Katherine A. and Davis Frank M. (both from the purple cluster in **Figure 3**) have made a considerable contribution to this field in recent years, whose publications were all published in the last five years with higher total citations per year (**Figure 4**).

### 3.3 Collaboration map of countries and institutes

**Figure 5:**
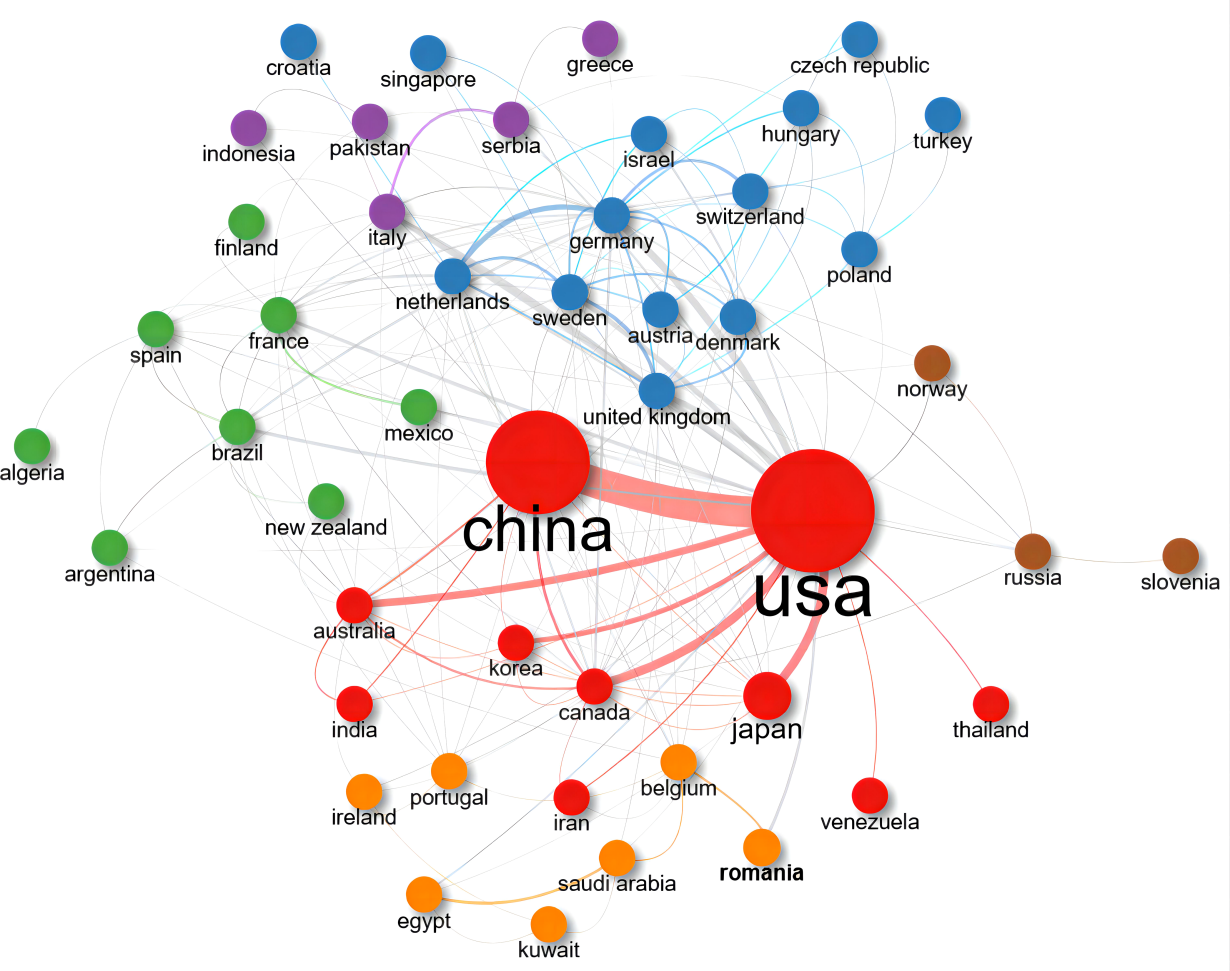
Collaboration network map of the 50 top-publishing countries between 2000 and 2022. Nodes represent different countries, colors represent clusters, and lines represent connections between them. Cluster 1: red (10 items); Cluster 2: blue (14 items); Cluster 3: green (8 items); Cluster 4: orange (7 items); Cluster 5: purple (5 items); Cluster 6: brown (3 items). Three isolated nodes were removed.

**Figure 6:**
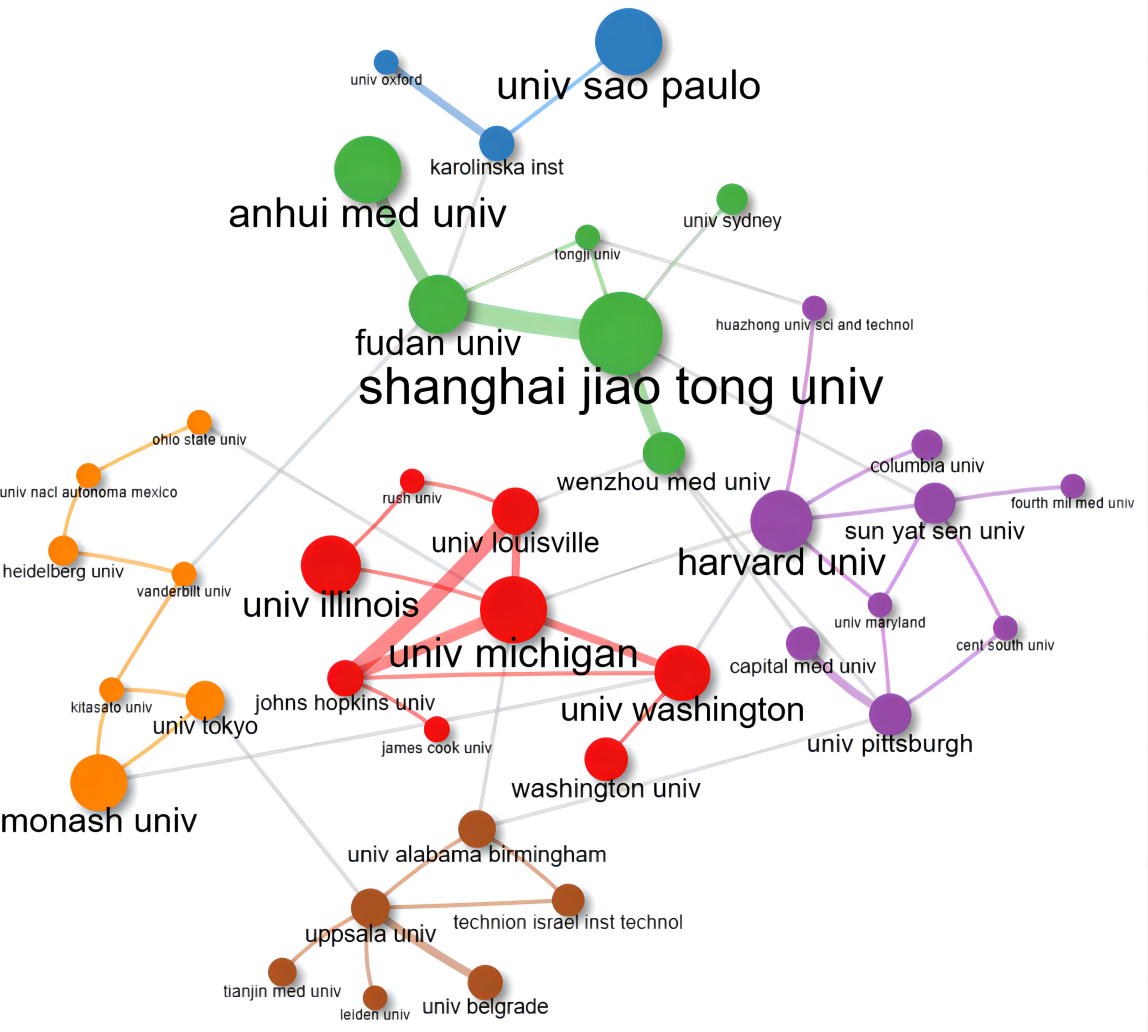
Collaboration network map of the 50 top-publishing institutes between 2000 and 2022. Nodes represent different institutes, colors represent clusters, and lines represent connections between them. Cluster 1: green (6 items); Cluster 2: red (8 items); Cluster 3: purple (9 items); Cluster 4: orange (7 items); Cluster 5: brown (6 items); Cluster 6: blue (3 items).11 isolated nodes were removed.

We selected the top 50 countries and institutes that ranked according to the number of publications. The PageRank score of each country or institute was calculated through their connections to others. **Figure 5** and **Figure 6** showed cooperative network maps of countries and institutes, respectively. From the perspective of national cooperation, the closest academic connection was observed between China and the United States of America. The blue nodes formed the largest cluster (14 countries), centered on Germany (PageRank score: 0.077) and mainly composed of European countries.

We listed the top ten high-producing countries and institutes according to the number of papers published and PageRank scores (**Table 2**). China was the leader in this field with 315 publications, followed by other top-publishing countries, including the United States of America (293 publications) and Japan (123 publications). The institute with the most published papers was the University of Michigan (75 publications). According to the PageRank scores, the United States of America was the most influential country, and Shanghai Jiao Tong University was the most significant research institute.

**Table 2:** Top 10 most influential countries and institutions.

Items	Publications			PageRank		
	Ranking	Name	Number	Ranking	Name	Score
Countries						
	1	China	315		USA	0.242
	2	USA	293		Germany	0.077
	3	Japan	123		China	0.075
	4	Australia	51		Sweden	0.049
	5	Germany	47		Canada	0.048
	6	Brazil	43		United Kingdom	0.042
	7	Canada	36		Netherlands	0.041
	8	France	35		France	0.034
	9	Korea	29		Italy	0.034
	10	India	23		Australia	0.034
Institutions						
	1	Univ Michigan	75		Shanghai Jiao Tong Univ	0.100
	2	Washington Univ	64		Fudan Univ	0.100
	3	Univ Sao Paulo	56		Univ Michigan	0.091
	4	Sichuan Univ	55		Johns Hopkins Univ	0.086
	5	Shanghai Jiao Tong Univ	45		Univ Louisville	0.075
	6	Univ Illinois	44		Capital Med Univ	0.071
	7	Harvard Univ	38		Univ Pittsburgh	0.071
	8	Anhui Med Univ	37		Univ Belgrade	0.071
	9	Shandong Univ	32		Uppsala Univ	0.071
	10	Columbia Univ	29		Karolinska Inst	0.071

### 3.4 Influential journals overview

**Figure 7:**
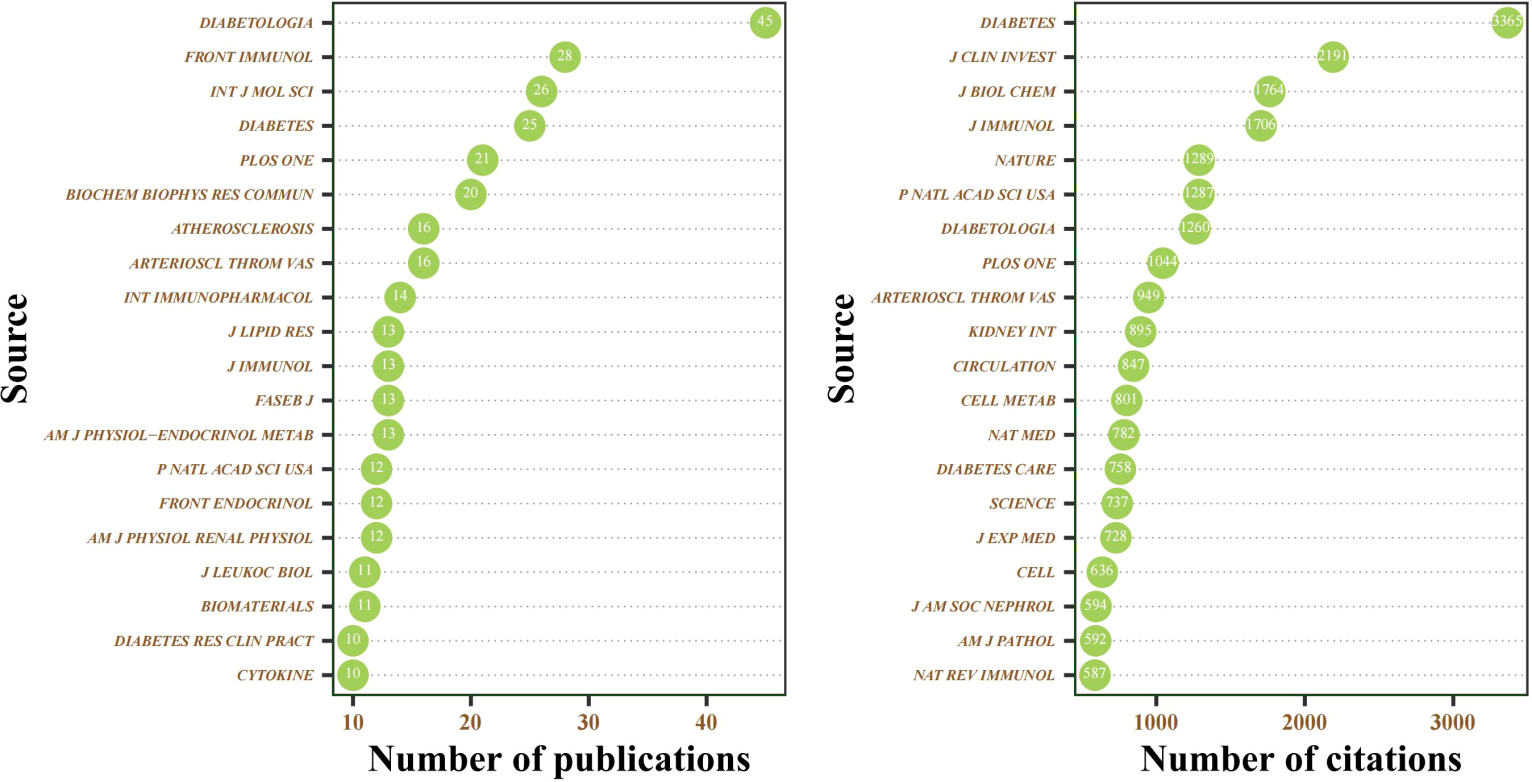
Cleveland dot plot of the top 20 journals ranked by publications and citations between 2000 and 2022.

All the included publications of the MRDS between 2000 and 2022 were published in 537 journals. According to the 2021 edition of the JCR, seven of the ten most highly published journals were in the Q1 JCR division, and two had an impact factor greater than 10 (**Supplementary Table 1**). As depicted in **Figure 7** and **Supplementary Table 1**, *Diabetologia* (publication:45; IF:10.460), *Frontiers in Immunology* (publication:28; IF:8.787), and *International Journal of Molecular* (publication:26; IF:6.208) were the journals that published the most relevant publications. Additionally, *Diabetes* (citation:3365; IF:9.305), *Journal of Clinical Investigation* (citation:2101; IF:19.477), and *Journal of Biological Chemistry* (citation:1764; IF:5.485) were journals with the highest number of citations.

### 3.5 Top-cited publications

**Supplementary Table 2** summarized top-cited papers to reveal essential publications from our bibliometric dataset. To better show the citation trend in relatively recent years, we ranked the top ten top-cited publications between 2000 and 2022 in terms of both total citations and total citations per year, respectively. The top-cited paper with the highest total citations was “Adipocyte Death Defines Macrophage Localization and Function in Adipose Tissue of Obese Mice and Humans”, an original full-length research article that published by Saverio Cinti et al., and “Inflammation as a Link Between Obesity, Metabolic Syndrome and Type 2 Diabetes” by Nathalie Esser et al. had the highest total citations per year. Notably, a review published in 2017 named “Macrophage Phenotypes Regulate Scar Formation and Chronic Wound Healing” (**Supplementary Table 2**) was the most recent highly cited publication according to total citations per year.

### 3.6 Visual analysis of research hotspots

**Figure 8:**
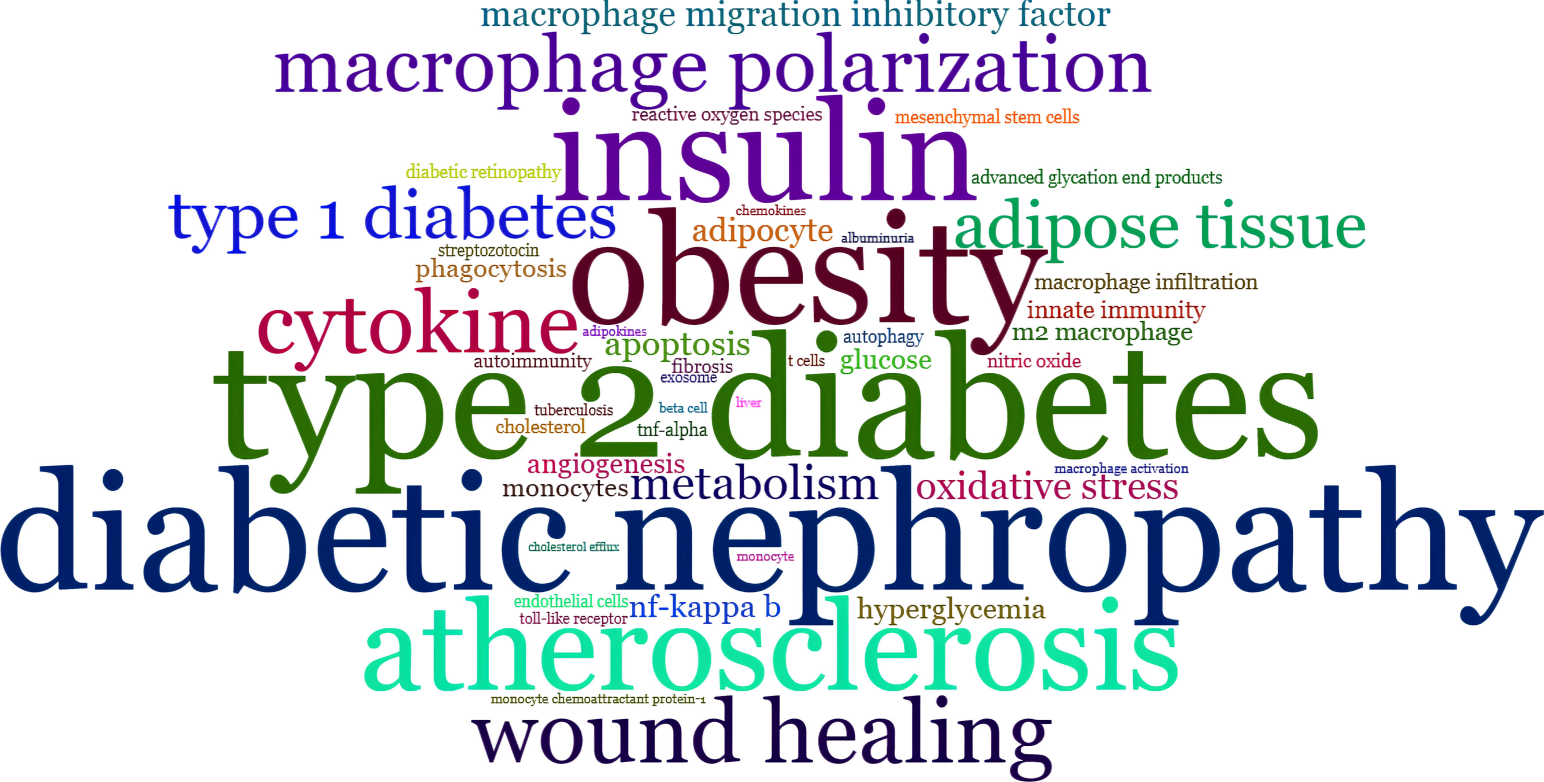
Word-cloud map of top 50 authors’ keywords ranked by frequency of occurrence in publications between 2000 and 2022. The font size represents the frequency of occurrence.

**Figure 9:**
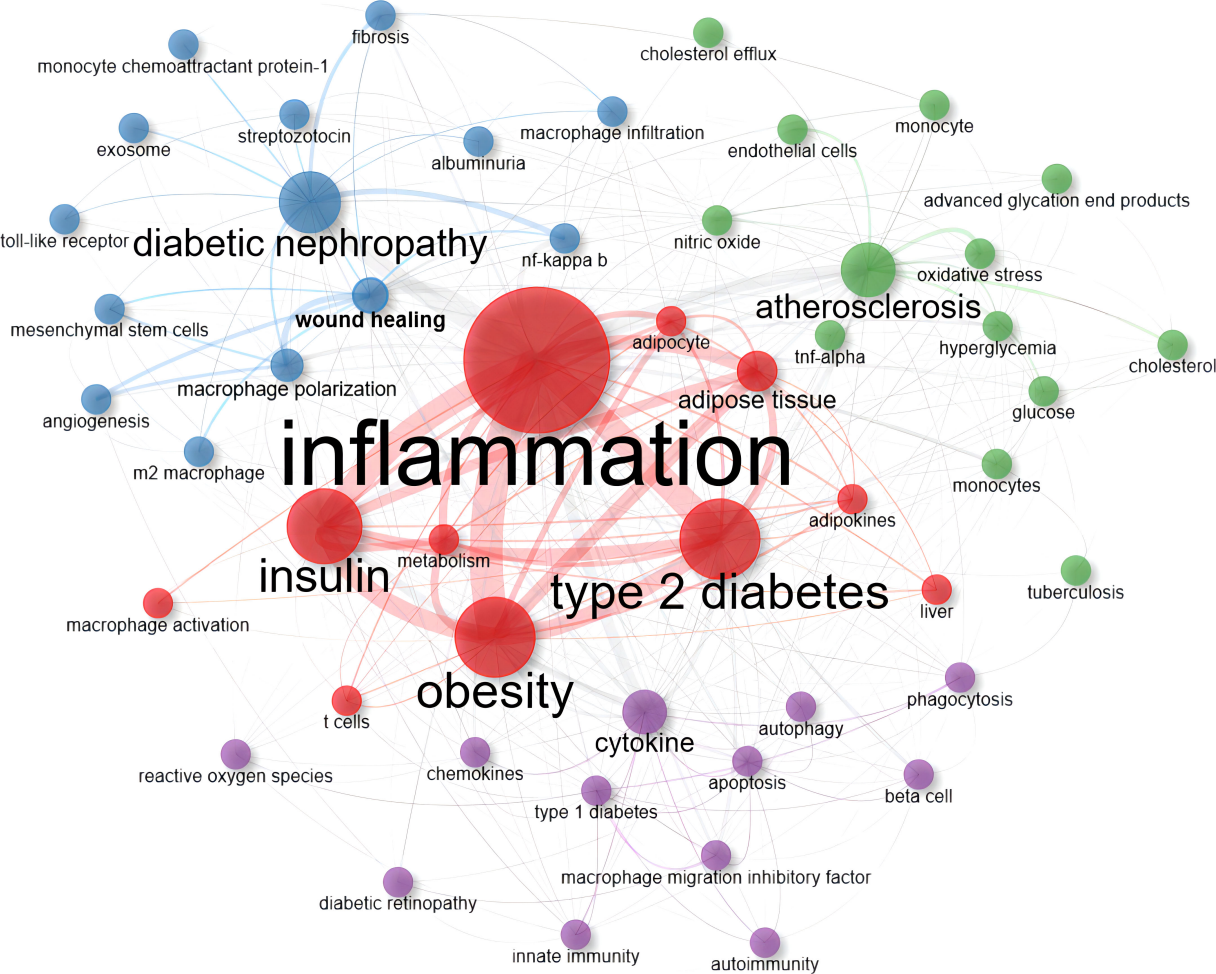
Co-occurrence network map of top 50 authors’ keywords ranked by frequency of occurrence in publications between 2000 and 2022. Nodes represent different keywords, colors represent clusters, and lines represent connections between them. Cluster 1: red (11 items); Cluster 2: blue (14 items); Cluster 3: green (13 items); Cluster 4: purple (12 items). None isolated nodes were detected.

Keywords are well-suited to be selected for exploring research hotspots and trends as they are typically high-level summaries of the publication. In the current study, we identified 2495 authors’ keywords. In order to facilitate co-occurrence visualization, we extracted the top 50 keywords by occurrence frequency and calculated the PageRank score between them. As shown in **Figure 8** and **Supplementary Table 3**, in the exclusion of search terms such as “diabetes” and “macrophage”, the keywords with higher occurrences were “inflammation” (277 times), “diabetic nephropathy” (123 times), “obesity” (114 times), and “insulin” (105 times). These authors’ keywords appeared more than 100 times, indicating their core position in this research field. In **Figure 9**, all closely related keywords were automatically grouped into four clusters and displayed in different colors. The red one, which was composed of “inflammation”, “metabolism”, “insulin” and “obesity”, was the most closely linked cluster. Among them, the term “inflammation” (PageRank: 0.183) almost intersected with all keywords that appeared in **Figure 9**, which undoubtedly represents it was the most exciting hotspot in this research field.

### 3.7 Temporal distribution map of forefront research topics

**Figure 10:**
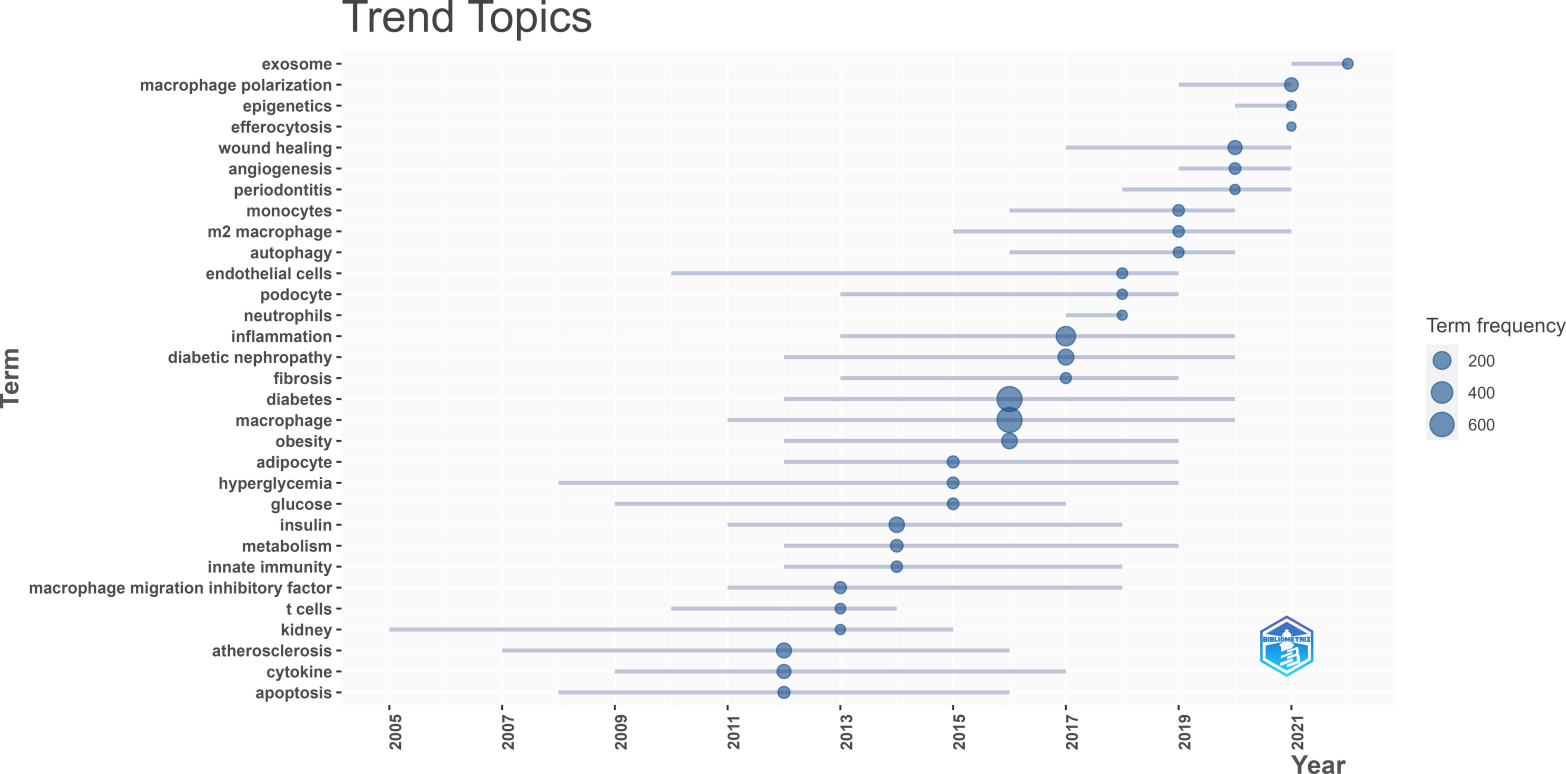
Trend topics map of Author’s Keyword for publications published between 2012 and 2022. Data and visualization were generated from the Documents menu of the Bibliometrix R-package.

After exploring the hotspots in MRDS, we made a further effort on the trend topic analysis based on keywords in combination with the evolution of time to reveal the current and promising forefront research topics. In our study, we identified the authors’ keywords from papers published from 2012 to 2022 that appear at least five times a year. Among them, a maximum of three themes were selected as annual topics. Precisely, 31 buzz topic terms of the year were calculated. The topic term at the top of **Figure 10** was “exosome” which was followed by “macrophage polarization” and “epigenetics”, denoting the current motor topics with potential research prospects. While the annual topics at the bottom, such as “atherosclerosis”, “cytokine” and “apoptosis” have been studied in the past with relatively mature development. Therefore, these topics which were listed as research hotspots in **Supplementary Table 3** and have not been calculated as forefront research topics.

**Figure 11:**
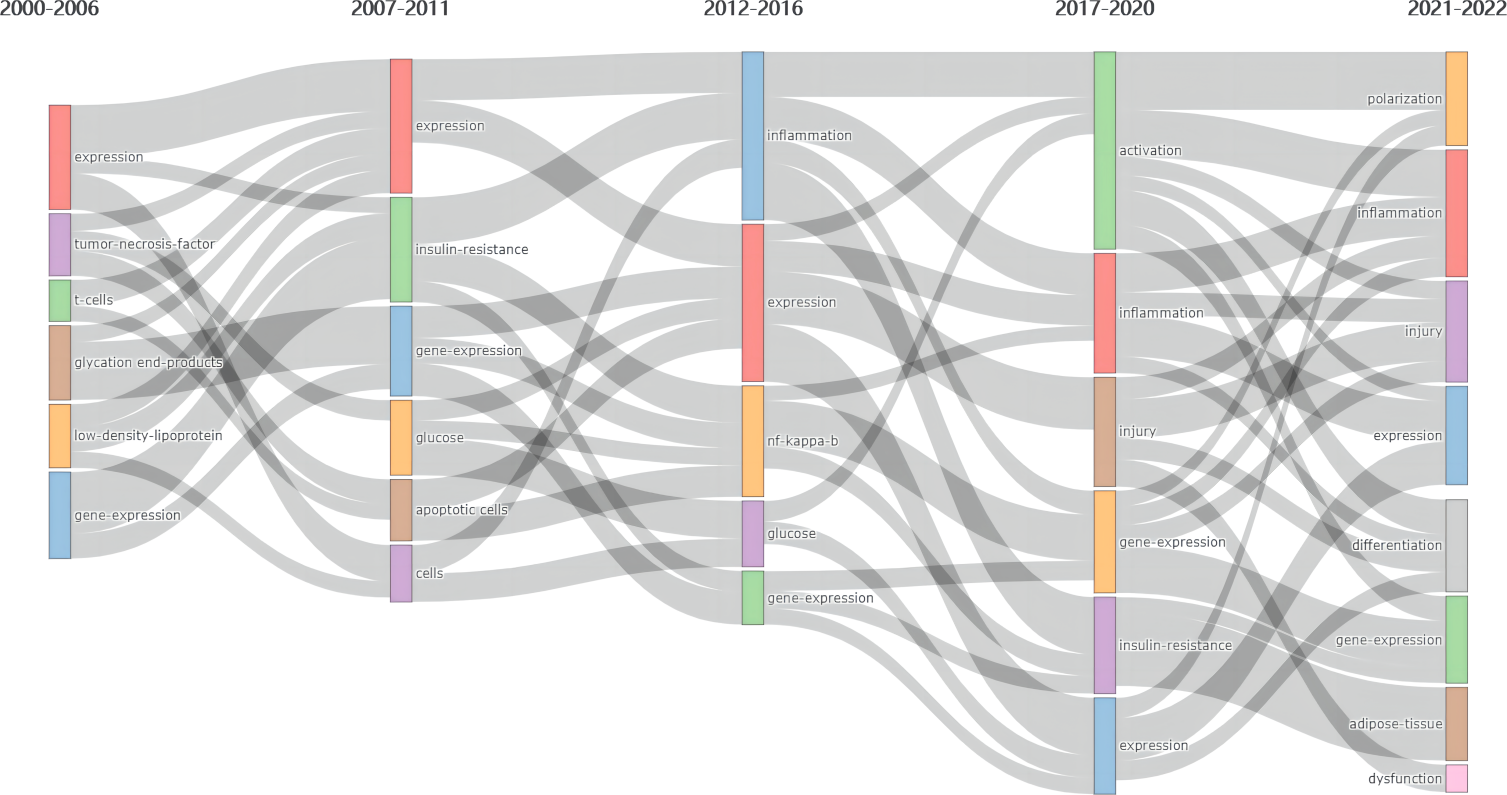
Thematic evolution map of Keyword Plus^®^ in publications published between 2000 and 2022. Data and visualization were generated from the Conceptual Structure menu of the Bibliometrix R-package. Column 1 (2000-2006): expression, tumor necrosis factor, t cells, glycation end products, low density lipoprotein, gene expression; Column 2 (2007-2011): expression, insulin resistance, gene expression, glucose, apoptotic cells, cells; Column 3 (2012-2016): inflammation, expression, nf kappa b, glucose, gene expression; Column 4 (2017-2020): activation, inflammation, injury, gene expression, insulin resistance, expression; Column 5 (2021-2022): polarization, inflammation, injury, expression, differentiation gene expression, adipose tissue, dysfunction.

Keyword Plus^®^ are terms frequently appearing in the titles of a publication’s references, automatically extracted by Web of Science *via* computer algorithms, and have been confirmed with more broadly descriptive (21). Hence, using Keyword Plus^®^ to study the change in research trends from different periods is appropriate and objective. As shown in **Figure 11**, we performed a thematic evolution map to display the development of MRDS based on summarizing various evolutionary associations *via* Keyword Plus^®^. To better reflect the research focus and trends, we divided the collection of publications from the past two decades into five periods: 2000-2006, 2007-2011, 2012-2016, 2017-2020, and 2021-2022. Throughout these five periods, the term “expression” and “gene expression” have been the core theme of such research, while the term “inflammation” became an emerging theme in the third period (2012- 2016), and it is still a research topic in great demand up to now. In the last two years, the fifth period, four new themes have emerged, including “polarization”, “differentiation”, “adipose-tissue” and “dysfunction” which shows the evolution of themes in MRDS has been ongoing and continues to progress.

## 4 Discussion

To our knowledge, this is the first bibliometric analysis to reveal the structural associations and temporal dynamics in macrophage research related to the diabetes field. Our objective was to conduct a comprehensive bibliometric analysis of the evolution of this field over the past two decades using the Bibliometrix R-package. Through visualized results, the quantitative data on authors, institutes, countries, sources, and keywords provided valuable insights into research hotspots and trends.

### 4.1 Bibliometric information

The annual publication analysis of 1272 papers published between 2000 and 2022 has shown a steady increase on the whole. After a long phase of knowledge accumulation from 2000 to 2011, identified as the beginning period, MRDS has entered a growth trend since 2012, especially in the past five years. The intense interest in macrophage function in influencing the process and treatment of diabetes reached its peak in 2021. Notwithstanding that our publication was conducted before the end of 2022, the fitting curves in **Figure 2** indicated that this popular trend would continue. Macrophage studies were expected to become a future direction and offer enlightening insight into the general landscape of the diabetes domain.

Regarding research productivity by authors, Wu Yonggui, the most prolific scholar with the highest H-index (23 publications, H-index:12) from Anhui Medicine University, mainly contributed to how herbal extracts regulate the pathological process of diabetic nephropathy (DN) through macrophages (22). The geographical distribution of this field was obtained by exploring productive countries and institutes, which could reflect their degree of influence and research skills. Chinese scholars published the most papers (315 publications), followed by American scholars with 203 publications. This situation might be related to the fact that China has a sizeable population with diabetes, and thus extra emphasis is placed on diabetes-related studies at the bench and clinical (23). As expected, five of the top ten high-yield research institutes were located in the United States of America, and four in China, reflecting the core position of these two countries in this field.

Based on the PageRank algorithm, we visualized the cooperation and influence of authors, countries, and institutes, respectively. This newly emerged bibliometric methodology can more objectively reflect the weight of items through the co-occurrence times (20). The most robust academic collaboration was observed between China and the United States of America in **Figure 5**. Although Chinese scholars published the most papers on macrophage studies related to diabetes, the United States of America was located in the most central position in this field, which also illustrates, to some extent, that Chinese scholars should pay more attention to international academic collaboration with other countries to break down knowledge barriers. However, no apparent core authors and institutes were observed in **Figure 3** and **Figure 6** due to no evident difference in PageRank scores. It is worth noting that there still needs to be more in-depth cooperation and communication among these active scholars and institutes.

When evaluating the significance of a publication, it is common to consider the number of citations it receives. Highly-cited papers usually serve as the foundation and framework of the field. Our study enumerated the top ten highly cited papers according to total and annual citations, respectively. The top-cited paper, a full-length original research article ranked by total citations, mainly discussed how macrophage infiltration in white adipose tissue is associated with the metabolic complications of obesity (24). Interestingly, the top-ranked publication by the total citations per year also reviewed the association of inflammation, represented by macrophage infiltration, with obesity, metabolic syndrome, and diabetes (25). The link between inflammation and metabolic disease is a hot topic in macrophage research.

### 4.2 Detection of research hotspots

The decrease in insulin-stimulated glucose uptake, known as insulin resistance, is an essential antecedent pathophysiological feature and a significant underlying etiology of T2D (5). Several interrelated mechanisms have been proposed to explain this process, all of which are associated with inflammation or the induction of an inflammatory response. These mechanisms include glucotoxicity, lipotoxicity, oxidative stress, endoplasmic reticulum stress, etc. (2–4). Metabolic disorders, such as obesity and dyslipidemia, increase the risk of overt T2D and its progression through the activation immune system and secondary chronic low-grade inflammation (26). Hence, inflammation is the pathogenic clue that links obesity, insulin resistance, and T2D (27). This association can be observed in the red cluster of **Figure 9**, and the central position of inflammation in the macrophage research related to diabetes is also well illustrated in **Figure 8**.

Pro-inflammatory macrophages, as a primary effector cell type, play a role in this phenomenon by its recruitment, accumulation, and activation in the insulin target tissue, in particular adipose tissue, upregulating the production of adipose-derived pro-inflammatory cytokines (25, 28). Adipose tissue is regarded as a highly specialized tissue that exerts endocrine functions based on autocrine and paracrine activities. Instead of solely storing energy, it synthesizes and secretes adipokines (29). Moreover, Adipose tissue inflammation is an important event in obesity and T2D, negatively affecting the transmission of insulin signals, which is mainly attributed to the pro-inflammatory effect of adipose tissue macrophages. Macrophages account for approximately 10% of adipose tissue in lean mice, while in obese and leptin-deficient mice, this proportion increases to more than 50% (30, 31). Additional studies have shown that preventing macrophage accumulation in adipose tissue or pro-inflammatory macrophage signaling can improve glucose metabolism and alleviate insulin resistance in obese mice (32, 33). The major mechanistic evidence can be ascribed to the inflammatory modifications by the enhanced secretion of pro-inflammatory cytokine mediated by macrophages. Hotamisligil et al. found elevated gene expression levels of TNF-α in adipose tissue of insulin resistance and obese rodents (34). In the following years, macrophage-derived TNF-α was identified as the major source (35, 36). Higher TNF-α concentrations in plasma result from macrophage accumulation, which significantly affects the regulation of insulin sensitivity (29, 37).

Kidney inflammation is a critical factor in the pathogenesis of DN, which is characterized by excessive extracellular matrix deposition (38). In the context of DN, elevated glucose levels and metabolites contribute to cellular inflammation, glomerular sclerosis, and tubulointerstitial fibrosis (39). The role of macrophages in the pathogenesis of DN has attracted mounting attention. Specifically, the activation and infiltration of macrophages in this process play a crucial role in promoting renal inflammation and fibrosis in both glomeruli and the tubulointerstitium (40, 41). Macrophage infiltration has been confirmed to be responsible for multiple pathological changes, including renal interstitial proliferation and irreversible pathological changes in glomeruli, aggravating the development of DN (42–44). The NADPH oxidase Nox4 is an isoform of the catalytic subunit of NADPH oxidase and is primarily responsible for generating renal reactive oxygen species in rodent models of DN induced by streptozotocin. Removing Nox4 can reduce glomerular macrophage infiltration and provide renal protection against glomerular injury (45).

Additionally, it is worth mentioning that the green cluster in **Figure 9** reflects metabolic disease augmentation on vascular disease mediated by macrophages and common mechanisms underlying atherosclerosis, diabetes, and obesity. As the primary cause of coronary artery disease, a common comorbidity of obesity and diabetes, atherosclerosis is a chronic inflammatory disease that has received considerable attention (46). Compared with purely inflammatory vascular disease individuals, macrophages from patients with coronary artery disease show significantly higher levels of glucose utilization and expression of pro-inflammatory cytokines, particularly TNF-α (47). Macrophage substrate metabolism is sensitive to extracellular glucose and lipid levels due to hyperglycemia further promotes lipid accumulation and lipotoxicity by increasing the expression of long-chain acyl CoA synthetase-1, and this glycemic condition could also lead to protein glycosylation and formation of advanced glycation end products producing massive pro-inflammatory cytokines (48, 49).

### 4.3 Future direction and breakthrough

The classical view of the macrophage polarization model describes two opposite phenotypic states: pro-inflammatory M1-like macrophages, the classic phenotype, which can be triggered by bacterial lipopolysaccharide, interferon-γ, and secret several pro-inflammatory cytokines including TNF-α, IL-1β, IL-6. In contrast, M2-like macrophages, the alternative phenotype induced by IL-4 and IL-13, play a role in anti-inflammation by producing IL-10 (50, 51). Multiple stimulating factors determine the phenotype of macrophages, and new subtypes are also being discovered (31). Although it is too polarized to generalize different subtypes of macrophages through highly simplified phenotype dichotomy, we mainly mentioned macrophages as either M1 or M2 for clarity purposes in this work.

Epigenetics, a fast-growing research field, is currently defined as an alteration in gene function in response to external stimuli that modify gene expression with no change in DNA sequence *via* DNA methylation, histone modifications, and non-coding RNAs (51). Due to the remarkable plasticity of macrophages, M1/M2 polarization is a dynamic process that can be reversed under different pathophysiological conditions and is associated with epigenetic modification (52). The predominance of these phenotypically distinct macrophages at specific times plays different roles. **Figure 10** and **Figure 11** showed that macrophage polarization could be a breakthrough point in this field. Here we discuss the current understanding of macrophage polarization in diabetic complications.

Wound healing involves four classical phases: hemostasis, inflammation, proliferation, and remodeling. This complex and well-coordinated process is promoted by multiple factors, among which the convergence of macrophages at the wound site is a decisive step in the healing process (53, 54). Specifically, the early inflammatory phase is dominated by the M1 macrophages, which are responsible for events such as bacterial phagocytosis, tissue debris, and the release of cytokines (e.g., TNF-α, IL-1β, nitric oxide synthase, IL-6) (55). Over time, the M2-like macrophage subtype becomes dominant, triggering the proliferative phase. The function of M1-like macrophages transforming into anti-inflammatory phenotype plays a vital role during normal wound repairment (51). However, under the influence of diabetic circumstances, this transition is impeded, leading to excessive wound inflammation and problematic wound resolution (56–58). Diabetic wounds tend to experience a higher presence of M1 macrophages, which can lead to delayed healing (59). Gallagher Katherine A. from the University of Michigan, USA, who made outstanding contributions to diabetic wound repair in the past five years (**Figure 4**), reversed the inflammatory macrophage phenotype and alienated wound repair by inhibiting the cyclooxygenase 2/prostaglandin E2 pathway (60).

As mentioned, macrophages are regarded as an indispensable source of TNF-α. Increased TNF-α levels in the kidney have been well-validated in models of DN (61). To support this, recent evidence has pointed out that compared with control mice, conditional ablation of TNF-α in macrophages significantly reduced albuminuria, alleviated the increase of plasma creatinine and blood urea nitrogen, and improved histopathological changes (62). M1 is the primary macrophage type located at the site of diabetic renal injury, producing inflammatory cytokines, such as IL-1β and TNF-α, which are aggravated by hyperglycemia (10). More importantly, the number of M1 macrophages that infiltrated the kidneys of mice with diabetes could gradually increase over time (63). Evidence from experimental models of diabetes also has shown a positive correlation between increased M1 polarization and renal injury (64). However, M2 therapies would become profibrotic at some point because M2 macrophages could release high levels of transforming growth factor-β1 to enhance kidney fibrosis (65). Although the exact M2 repolarization phenotype remains to be further elucidated, inhibiting the activation of M1 macrophages and promoting the polarization of M2 macrophages have the potential to prevent podocyte damage, reduce proinflammatory cytokines, and reduce profibrotic proteins against DN (66–69).

Initiation and maintenance of atherosclerosis (AS) are well acknowledged as a chronic inflammatory state of the arterial wall in which several immune cells participate, and atherosclerotic plaque formation and progression are highly associated with the epigenetic regulation of macrophages, particularly macrophage-produced cytokines (70, 71). According to histological analysis of human atherosclerotic plaques, compared with M2 macrophages, M1 macrophages were most located in lipid-enrich areas. *In vivo*, the increase of M1 macrophages could accelerate the progression of AS contributing to necrotic core formation, plaque rupture, and coagulation cascade activation. In contrast, the rise in M2 macrophage polarization could suppress this process (72, 73).

Patients with diabetes have a high risk of complicating coronary artery disease, which leads to more diffuse AS under the influence of increased infiltration of inflammatory macrophages (74). Macrophages can be polarized toward the M1 phenotype within the atherosclerotic plaque in response to the exposition of various oxidized derivatives and growth factors. As a representative pathological toxic metabolite of diabetes, advanced glycation end products could trigger macrophages to polarize toward M1 by interacting with its receptors *via* RAGE/TLR4 signaling, leading to the inducement and aggravation of AS, and specifically inhibiting the activation of TLR4 signaling could effectively reduce plaque M1 macrophage infiltration and arterial stenosis (70, 75). Another key finding was that the overexpression of brain-derived neurotrophic factors (BDNF) could promote the M2 macrophage polarization to alleviate diabetes-related AS (76). As a critical regulator of the survival, differentiation, and growth of neurons, expression of BDNF is observed in activated macrophages and promotes the transformation of macrophages from M1 to M2 (77). In the acute phase of human coronary artery AS, the plasma level of BDNF decreases, which can also be observed in individuals with diabetes (78, 79). Taken together, it reflects diabetes augmentation on vascular disease mediated by macrophages. Promoting the polarization of M1 macrophages to M2 can be a breakthrough to better interpret and alleviate inflammatory diseases, including diabetes-related conditions.

Notably, exosomes, a type of cell vesicle with lipid bilayer membranes containing functional protein and nucleic acids, have been attractive in macrophage-related diabetes studies (**Figure 9**). As mentioned, macrophages are the predominant immune cells, and their polarization can mediate the inflammatory process. In this scenario, exosomes can further control the inflammatory conditions in various inflammatory diseases by regulating the differentiation of macrophages (80). Through this mechanism, exosomes which were originated from stem cells transferred into macrophages to induce the anti-inflammatory M2 macrophage polarization through the transactivation of arginase-1 with remarkably increased anti-inflammatory factors including IL-10, but no significant changes in those of M1-related cytokines which in turn evoked inflammatory responses and enhanced insulin sensitivity (81). The development of neuronal injury in rats could be accelerated by the miR−21−5p, carried by exosomes derived from dorsal root ganglia neurons, inducing microglia, which served as brain macrophage, differentiated into the proinflammatory M1 phenotype (82). Lv et al. found that high levels of miR-19b-3p positively correlated with the severity of tubulointerstitial inflammation in patients with DN and demonstrated that exosomal miR-19b-3p mediated the communication between injured tubular epithelial cells and macrophages, leading to M1 macrophage activation and further tubulointerstitial inflammation (83). In addition, exosomes secreted from human serum albumin−treated HK−2 cells released miR−199a−5p, which targeted the TLR4 pathway to induce M1 polarization, resulting in the progression of DN (84).

Admittedly, our work has some common limitations of bibliometric analysis. First, Boolean search terms (OR/AND) were performed to establish our bibliometric database by merging the publication information of diabetes and macrophage fields. However, this process would inevitably result in an amount of included literature that did not fit our research topic. To address this issue, we refined our search strategies and manually screened each publication record. Second, due to the limited number of publications included, co-citation analysis could not be conducted to obtain interpreted results. In addition, our research mainly focused on analyzing and mining quantitative metrics to describe the current state and temporal dynamics in MRDS systematically. However, qualitative analyses were not performed and the final results should be explained cautiously (85).

## 5 Conclusions

With the help of the bibliometrix R-package, a comprehensive bibliometric analysis was performed to visualize the temporal dynamics and research trends of the MRDS knowledge domain from 2000 to 2022. Overall, the annual quantity of publications has increased steadily. China, the University of Michigan, and the *Diabetologia* journal have the highest volume of publications compared to other countries, institutes, and journals. Our findings delineate the mainstream of MRDS mainly focused on inflammation and its related mechanisms. Macrophage polarization is currently the most cutting-edge topic. This pathophysiological mechanism has been explored in diabetes and its complications which has the potential as a fulcrum for future diabetes-related translational research. We aimed to provide valuable insights and clues for endocrinologists and academics to conduct further studies. Furthermore, the results of this study are also expected to strengthen investment policies in future macrophage-related diabetes research.

## Data availability statement

The original contributions presented in the study are included in the article/**Supplementary Material**. Further inquiries can be directed to the corresponding authors.

## Author contributions

LZhao and BZ designed and supervised the study. SW and LZhang wrote the first draft of the manuscript. ZJ and YW extracted the data, performed analyses, and interpreted the data. LZhao and BZ contributed to the polishing and revising the manuscript and resolved all discrepancies. All authors contributed to the article and approved the submitted version.
