# Supplementary material for: Visualizing temporal dynamics and research trends of macrophage-related diabetes studies between 2000 and 2022: a bibliometric analysis
Source: Front Immunol. 2023 Jul 26;14:1194738. doi: 10.3389/fimmu.2023.1194738 (PMC10410279; doi:10.3389/fimmu.2023.1194738)
Supplement: Supplementary file 2 [file Table_2.docx]

Supplementary Material

Visualizing Temporal Dynamics and Research Trends of Macrophage-Related Diabetes Studies between 2000 and 2022: a Bibliometric Analysis

Sicheng Wang^1¶^, Lili Zhang^1¶^, Zishan Jin^1,2^, Yayun Wang^1,3^, Boxun Zhang^1*^, and Linhua Zhao^1*^

¶ represents that these authors contributed equally.

*** Correspondence:**Boxun Zhang: 1243876560@qq.com;

Linhua Zhao: [melonzhao@163.com](mailto:melonzhao@163.com).

# Supplementary Tables

**Supplementary Table 1** Top ten journals contributed to publications between 2000 to 2022.

| **Ranking** | **Journal** | **Count** | **JCR division** | **Impact Factor (2021)** | **Category** |
| --- | --- | --- | --- | --- | --- |
| 1 | *Diabetologia* | 45 | Q1 | 10.460 | Endocrinology & Metabolism |
| 2 | *Frontiers in Immunology* | 28 | Q1 | 8.787 | Immunology |
| 3 | *International Journal of Molecular Sciences* | 26 | Q1 | 6.208 | Biochemistry & Molecular Biology |
| 4 | *Diabetes* | 25 | Q1 | 9.305 | Endocrinology & Metabolism |
| 5 | *PLoS One* | 21 | Q2 | 3.752 | Multidisciplinary Sciences |
| 6 | *Biochemical And Biophysical Research Communications* | 20 | Q3 | 3.322 | Biochemistry & Molecular Biology |
| 7 | *Arteriosclerosis Thrombosis and Vascular Biology* | 16 | Q1 | 10.514 | Hematology |
| 8 | *Atherosclerosis* | 16 | Q1 | 6.851 | Cardiac & Cardiovascular Systems |
| 9 | *International Immunopharmacology* | 14 | Q2 | 5.714 | Immunology |
| 10 | *American Journal of Physiology-Endocrinology and Metabolism* | 13 | Q1 | 5.900 | Endocrinology & Metabolism |

**Supplementary Table 2** Top ten highly cited publications between 2000 and 2022.

| **Ranking** | **Ranked by TC per year** | | | | **Ranked by TC** | | | |
| --- | --- | --- | --- | --- | --- | --- | --- | --- |
|  | **Paper (first author, year, PT)** | **DOI** | **TC per Year** | **TC** | **Paper (first author, year, PT)** | **DOI** | **TC per Year** | **TC** |
| 1 | Esser N, 2014, review. | 10.1016/j.diabres.2014.04.006 | 114.44 | 1030 | Cinti S, 2005, article. | 10.1194/jlr.M500294-JLR200 | 91.39 | 1645 |
| 2 | Cinti S, 2005, article. | 10.1194/jlr.M500294-JLR200 | 91.39 | 1645 | Bastard JP, 2006, review. | NA; PubMed ID: 16613757 | 87 | 1479 |
| 3 | Bastard JP, 2006, review. | NA; PubMed ID: 16613757 | 87 | 1479 | Esser N, 2014. | 10.1016/j.diabres.2014.04.006 | 114.44 | 1030 |
| 4 | Hesketh M, 2017, review. | 10.3390/ijms18071545 | 52.33 | 314 | Antunapuente B, 2008, review. | 10.1016/j.diabet.2007.09.004 | 34.67 | 520 |
| 5 | Johnson AR, 2012, review. | 10.1111/j.1600-065X.2012.01151.x | 36.10 | 397 | Ehses JA, 2007, article. | 10.2337/db06-1650 | 32.375 | 518 |
| 6 | Antunapuente B, 2008, review. | 10.1016/j.diabet.2007.09.004 | 34.67 | 520 | Sun QH, 2009, article. | 10.1161/CIRCULATIONAHA.108.799015 | 33.57 | 470 |
| 7 | Sun QH, 2009, article. | 10.1161/CIRCULATIONAHA.108.799015 | 33.57 | 470 | Silswal N, 2005, article. | 10.1016/j.bbrc.2005.06.202 | 24.56 | 442 |
| 8 | Ehses JA, 2007, article. | 10.2337/db06-1650 | 32.38 | 518 | Wetzler C, 2000, article. | 10.1046/j.1523-1747.2000.00029.x | 17.48 | 402 |
| 9 | Khanna S, 2010, article. | 10.1371/journal.pone.0009539 | 30.08 | 391 | Johnson AR, 2012, review. | 10.1111/j.1600-065X.2012.01151.x | 36.10 | 397 |
| 10 | Chawla A, 2010, review. | 10.1161/CIRCRESAHA.110.216523 | 26.92 | 350 | Chow F, 2004, article. | 10.1111/j.1523-1755.2004.00367.x | 20.79 | 395 |

### **Note:** TC: Total Citations; DOI: Digital Object Unique Identifier.

**Supplementary Table 3** Top 50 authors’ keywords ranked by frequency of occurrence in publications of MRDS between 2000 and 2022.

| **Ranking** | **Keyword** | **Count** | **PageRank score** | **Ranking** | **Keyword** | **Count** | **PageRank score** |
| --- | --- | --- | --- | --- | --- | --- | --- |
| 1 | Inflammation | 277 | 0.183 | 26 | Fibrosis | 14 | 0.011 |
| 2 | Diabetic Nephropathy | 123 | 0.053 | 27 | Cholesterol | 14 | 0.008 |
| 3 | Obesity | 114 | 0.085 | 28 | Autoimmunity | 14 | 0.007 |
| 4 | Insulin | 105 | 0.083 | 29 | TNF-α | 13 | 0.011 |
| 5 | Atherosclerosis | 93 | 0.052 | 30 | Mesenchymal Stem Cells | 13 | 0.008 |
| 6 | Wound Healing | 67 | 0.026 | 31 | Diabetic Retinopathy | 13 | 0.007 |
| 7 | Cytokine | 65 | 0.041 | 32 | Autophagy | 13 | 0.01 |
| 8 | Macrophage Polarization | 60 | 0.023 | 33 | Advanced Glycation End Products | 13 | 0.004 |
| 9 | Monocyte | 51 | 0.031 | 34 | Streptozotocin | 12 | 0.008 |
| 10 | Adipose Tissue | 50 | 0.04 | 35 | Endothelial Cells | 12 | 0.006 |
| 11 | Metabolism | 36 | 0.029 | 36 | Tuberculosis | 11 | 0.006 |
| 12 | Oxidative Stress | 29 | 0.017 | 37 | Toll-Like Receptor | 11 | 0.007 |
| 13 | Macrophage Migration Inhibitory Factor | 27 | 0.01 | 38 | T Cells | 11 | 0.008 |
| 14 | Apoptosis | 26 | 0.018 | 39 | Exosome | 11 | 0.005 |
| 15 | Adipocyte | 25 | 0.023 | 40 | Macrophage Activation | 10 | 0.005 |
| 16 | Nf-Kappa B | 24 | 0.018 | 41 | Liver | 10 | 0.009 |
| 17 | Hyperglycemia | 22 | 0.016 | 42 | Chemokines | 10 | 0.011 |
| 18 | Glucose | 21 | 0.012 | 43 | Beta Cell | 10 | 0.007 |
| 19 | Angiogenesis | 21 | 0.012 | 44 | Albuminuria | 10 | 0.006 |
| 20 | Phagocytosis | 20 | 0.01 | 45 | Adipokines | 10 | 0.014 |
| 21 | M2 Macrophage | 19 | 0.005 | 46 | Il-1 Beta | 9 | 0.006 |
| 22 | Innate Immunity | 18 | 0.009 | 47 | High Glucose | 9 | 0.007 |
| 23 | Macrophage Infiltration | 16 | 0.008 | 48 | Cholesterol Efflux | 9 | 0.005 |
| 24 | Reactive Oxygen Species | 14 | 0.005 | 49 | Dendritic Cells | 8 | 0.004 |
| 25 | Nitric Oxide | 14 | 0.009 | 50 | Cardiovascular Disease | 8 | 0.008 |
